# Treponemal Infection in Nonhuman Primates as Possible Reservoir for Human Yaws

**DOI:** 10.3201/eid1912.130863

**Published:** 2013-12

**Authors:** Sascha Knauf, Hsi Liu, Kristin N. Harper

**Affiliations:** German Primate Center, Göttingen, Germany (S. Knauf);; Centers for Diseases Control and Prevention, Atlanta, Georgia, USA (H. Liu);; Columbia University Medical Center, New York, New York, USA (K.N. Harper)

**Keywords:** yaws, treponeme, World Health Organization, syphilis, pallidum, pertenue, nonhuman primates, eradication, bacteria, Africa, zoonoses

**To the Editor:** In 2012, the World Health Organization launched plans for a second campaign to eradicate the neglected tropical disease, yaws (*1*). The first campaign, conducted during the mid-20th century, was tremendously successful in terms of treatment and reduced the number of cases by 95%. However, it failed to eradicate the disease, and when local efforts to prevent new cases proved insufficient, yaws resurged in some areas. Comments on the new yaws eradication campaign have emphasized the need for sustained support and resources. Here we draw attention to an additional concern that could impede yaws eradication efforts.

The success of any eradication campaign depends on the absence of a nonhuman reservoir. Smallpox had no known animal reservoir, and polio and dracunculiasis (guinea worm disease), which are currently the focus of the World Health Organization eradication campaigns, also have none. By contrast, compelling evidence suggests that yaws exists in wild nonhuman primate populations residing in regions where humans are also infected (Figure).

**Figure:**
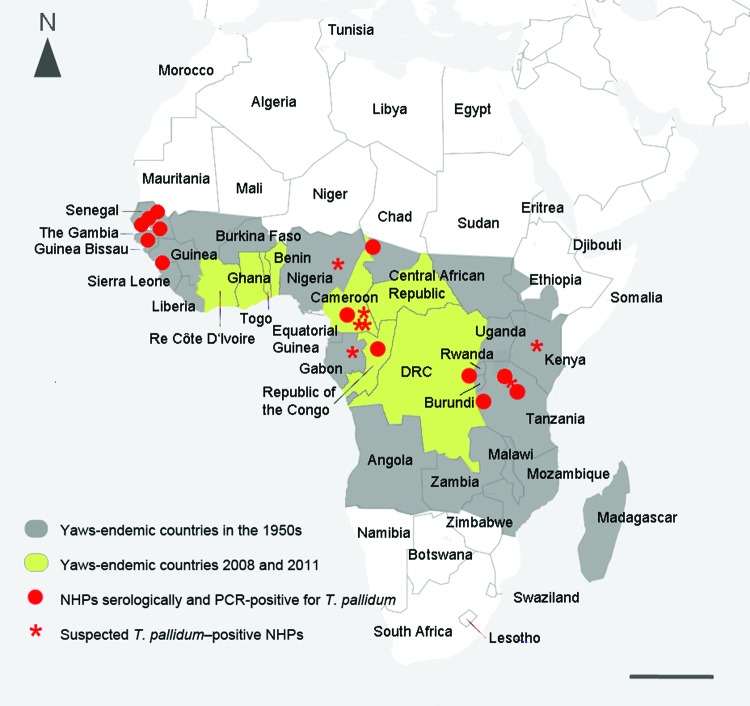
Geographic proximity between human yaws and endemic syphilis, as estimated by the World Health Organization, and locations in which treponemal infection has been identified in nonhuman primates (NHPs), Africa, 1990s. Red dots indicate infection in NHPs confirmed by sensitive and specific treponemal serologic tests (TPI/FTA-ABS/MHA-TP [Treponema-pallidum-immobilization reaction/fluorescence-Treponema-antibody-absorption test/Treponema pallidum microhemagglutination assay]) and, in some cases, PCR. Stars indicate suspected infection (i.e., sightings of NHPs with lesions consistent with infection). Sources include the following: 1) Cameroon: *Gorilla gorilla*, observation (W. Karesh, pers. comm.); *Pan troglodytes*, *G. gorilla*, and *Papio* sp., skeletal analysis and serology (*4*;*11* in Technical Appendix. 2) Chad: *Erythrocebus patas*, serology (*4*). 3) Democratic Republic of Congo (DRC): *Pan troglodytes*, serology (*4*). 4) Gabon: *G. gorilla*, observation (W. Karesh, pers. comm.). 5) Guinea: *Papio* sp., serology and PCR (*4,8*). 6) Kenya: *Papio anubis* and *Chlorocebus* sp., observation and serology (J. Fischer, pers. comm.); *12* in Technical Appendix). 7) Nigeria, *Papio anubis* (J. Wallis, pers. comm.). 8) Republic of Congo: *G. gorilla*, serology and observation (W. Karesh, unpub. data; *5*). 9) Tanzania: *P. anubis*; observation, serology, PCR (*6,7*; *13* in Technical Appendix; S. Knauf, unpub. data). 10) Senegal: *Papio* sp., *Chlorocebus* sp., colobus monkeys, and *Erythrocebus patas*; serology (S. Knauf, unpub. data; *4*; *14* in Technical Appendix). Scale bar = 1,000 km.

The subspecies of the bacterium *Treponema pallidum* that cause the non–sexually transmitted diseases yaws (subsp. *pertenue* infection) and endemic syphilis (subsp. *endemicum* infection) and the sexually transmitted infection syphilis (subsp. *pallidum*) are close relatives. The 3 diseases cannot be distinguished serologically. Instead, the diseases they cause are usually differentiated by clinical characteristics and geographic distribution. Whereas syphilis is a venereal disease with a worldwide distribution, yaws primarily affects children in hot and humid areas of Africa and Asia, and endemic syphilis occurs in arid regions. Because methods available to differentiate between the *T. pallidum* subspecies were unavailable in the past, prevalence data for yaws were sometimes vague and inaccurate. Recently, molecular tests capable of distinguishing between the subspecies by using single nucleotide polymorphisms have been developed (*2*,*3*). These tests have enabled us to learn more about the *T. pallidum* strains that infect wild nonhuman primates.

During the 1960s, researchers reported that many baboons in West Africa were seropositive for treponemal infection (*4*). Since then, high levels of infection have been documented in other monkey species in West Africa and in great apes (*5*). Recently, we documented *T. pallidum* infection in olive baboons (*Papio anubis*) at Lake Manyara National Park in Tanzania (*6*). In West Africa, clinical signs of infection in nonhuman primates are usually mild, if present at all, consisting of small lesions around the muzzle, eyelids, and armpits (*4*). A recent survey in 2013 at Parc National du Niokolo-Koba, Senegal, revealed *T. pallidum* antibodies in Guinea baboons (*P. papio*) with no signs of infection (S. Knauf et al, unpub. data). By contrast, severe manifestations resembling tertiary-stage yaws have been reported in wild gorillas (*5*). In terms of genetic distance, studies thus far indicate that the organisms infecting baboons in West and East Africa closely resemble *T. pallidum* subsp. *pertenue,* the agent responsible for yaws in humans (*2*,*7*). In fact, the genome sequence of a *T. pallidum* strain collected from a baboon in Guinea indicates that it should be considered a *T. pallidum* subsp. *pertenue* strain (*8*). Infection has been confirmed by serologic tests in a variety of nonhuman primate species in the yaws belt of Africa and by PCR in baboons from East and West Africa (Figure).

The high prevalence of nonhuman primate infection in areas of tropical Africa where yaws is common in humans (Figure) suggests that cross-species infection may occur. Decades ago, researchers reported that the Fribourg-Blanc simian strain, collected in Guinea, can cause sustained infection in humans after inoculation (*9*). Such experiments are ethically questionable and the details given are scant, but this work suggests that simian strains have zoonotic potential. Additional research is needed to determine whether interspecies transmission of *T. pallidum* occurs under natural conditions. Bush meat preparation is common in many African countries and a major source of zoonotic infection. It involves frequent skin-to-skin contact, which is the preferred mode of transmission for yaws. Insects also have been proposed to be vectors of infection, although this has not been documented (*10*). If evidence of interspecies yaws transmission, either direct or by vector, is discovered, then nonhuman primates may be a major reservoir of infection for humans.

Additional studies comparing human and simian strains may show whether zoonotic transmission of *T. pallidum* occurs frequently, an important consideration with regard to disease eradication and the conservation of great apes and other endangered nonhuman primates. To eradicate yaws, all host species and any possible reservoirs need to be taken into account. We, like the rest of the world, want the second yaws eradication campaign to succeed and hope that nonhuman primate infection will be evaluated as a factor in disease transmission.

Technical Appendix Additional references cited in the Figure.
