# Supplementary material for: Treponemal Infection in Nonhuman Primates as Possible Reservoir for Human Yaws
Source: Emerg Infect Dis. 2013 Dec;19(12):2058–60. doi: 10.3201/eid1912.130863 (PMC3840862; doi:10.3201/eid1912.130863)
Supplement: Technical Appendix — Additional references cited in the Figure. [file 13-0863-Techapp-s1.pdf]

Article DOI: http://dx.doi.org/10.3201/eid1912.130863

Treponemal Infection in Nonhuman Primates as Possible Reservoir for Human Yaws

Technical Appendix

Additional references cited in the Figure

11. Lovell N, Jurmain R, Kilgore L. Skeletal evidence of probable treponemal infection in free-ranging African apes. Primates. 2000;41:275–90. http://dx.doi.org/10.1007/BF02557597

12. Felsenfeld O, Wolf RH. Serological reactions with treponemal antigens in nonhuman primates and the natural history of treponematosis in man. Folia Primatol (Basel). 1971;16:294–305. PubMed http://dx.doi.org/10.1159/000155411

13. Wallis J, Lee DR. Primate conservation: the prevention of disease transmission. Int J Primatol. 1999;20:803–26. http://dx.doi.org/10.1023/A:1020879700286

14. Baylet R, Thivolet J, Sepetjian M, Bert J. Seroepidemiological studies on primate treponematosis in Senegal [in French]. Bull Soc Pathol Exot. 1971;64:836–41.
